# Cost-effective fibrinolytic enzyme production by *Bacillus subtilis* WR350 using medium supplemented with corn steep powder and sucrose

**DOI:** 10.1038/s41598-019-43371-8

**Published:** 2019-05-02

**Authors:** Rui Wu, Guiguang Chen, Shihan Pan, Jingjing Zeng, Zhiqun Liang

**Affiliations:** State Key Laboratory for Conservation and Utilization of Subtropical Agro-bioresources, Guangxi Microorganism and Enzyme Research Center of Engineering Technology; College of Life Science and Technology; Guangxi University, 100 Daxue Road, Nanning, 530004 Guangxi China

**Keywords:** Microbiology techniques, Biologics, Microbiology techniques, Biologics, Microbiology techniques

## Abstract

The goal of this study was to develop a cheap and simple medium and to optimize fermentation parameters for fibrinolytic enzyme production by *Bacillus subtilis* WR350. A low-cost medium containing 35 g/L sucrose, 20 g/L corn steep powder and 2 g/L MgSO_4_·7H_2_O was developed via single-factor and orthogonal experiments. A cheap nitrogen source, corn steep powder, was used to replace the soy peptone present in the initial medium. The highest fibrinolytic activity of 5865 U/mL was achieved using the optimized medium in a 100-L fermenter with an aeration rate of 1.0 vvm and an agitation speed of 200 rpm. The resulting enzyme yield was among the highest described in the literature with respect to fibrinolytic activity, as determined by the fibrin plate method. Techno-economic evaluation indicated that the cost of the optimized medium was only 8.5% of the cost of the initial medium, and the total fermentation cost of fibrinolytic enzyme production using the optimized medium was 23.35% of the cost of using the initial medium.

## Introduction

Cardiovascular diseases are the primary cause of death in human beings. Cardiovascular diseases, such as acute myocardial infarction, hypertension and stroke are typically attributed to the excessive accumulation of fibrin in blood vessels^1^. The emergence of thrombolytic drugs represents a turning point for this medical problem^2^. For thrombolytic therapy, the commonly used drugs involve tissue-typed plasminogen activator (t-PA), streptokinase (SK) and urokinase (UK). However, these agents have some limitations, such as their high costs and undesirable side effects, which include excessive bleeding and recurrence at the site of the residual thrombosis^3^. Therefore, studies have been conducted to enhance the efficacy and fibrin specificity of fibrinolytic enzymes, with microbial fibrinolytic enzymes having attracted a great deal of attention for use in medical treatments^4^. Microbial fibrinolytic enzymes have the potential to be developed as functional food additives and drugs to prevent or cure cardiovascular diseases. Various efforts have been devoted to the development of new types of fibrinolytic enzymes, with enzymes having been isolated from Indonesian traditional fermented foods^5^, *Cordyceps militaris*^6^, Chinese Douchi^7^, Japanese natto^8^ and food-grade *Bacillus subtilis*^4,9^.

Several studies have shown that *B. subtilis* produces a variety of fibrinolytic enzymes^10–12^. However, the high price of soy peptone and yeast extract currently used for fibrinolytic enzyme production has restricted its industrial application as a feedstock. Thus, in this study, the use of low-cost and easy-to-obtain nitrogen sources were investigated to reduce the cost of medium. Inexpensive inorganic nitrogen sources and agricultural byproducts have previously been used to replace soy peptone^13^. Corn steep powder (CSP) is a byproduct of the production of corn starch and can provide important nutrients (e.g., amino acids, proteins, vitamins, minerals and trace elements) for the growth of microbes^14^. More importantly, the price of industrial CSP is very cheap, and CSP has been used in place of soy peptone and yeast extract to produce many products, such as lactic acid^15^ and poly-γ-glutamic acid^14^. Nevertheless, the use of CSP to produce fibrinolytic enzymes has received little attention.

Environmental conditions are crucial to biomass and product formation, and process optimization for fibrinolytic enzyme production is typically performed using bench-scale bioreactors^16–18^. Nevertheless, the scale up of the process at a pilot fermenter scale has been seldom reported. Therefore, in the present study, after medium optimization in shake flasks, the operating conditions, such as agitation speed and aeration rate, were evaluated in a 100-L pilot fermenter^19,20^.

Among various operation conditions, oxygen supply (OS) is recognized as a significant factor for aerobic microbial fermentation^21^. Both agitation speed and aeration rate are important parameters for the control of OS and have a significant effect on the aerobic production of some biopolymers, including glycoprotein GP-1^20^ and bacterial cellulose^22^. Agitation exerts mixing and shearing effects in fermentation processes. Furthermore, agitation improves mass and oxygen transfer between different phases and can sometimes cause changes in strain morphology, variations in cell growth and metabolite formation. The aeration rate determines the oxygenation of the fermentation process and contributes to mixing of the fermentation medium, especially when mechanical agitation speeds are low^20,23^.

In this study, single factor experiments combined with an L_9_(3^4^) orthogonal design were performed to improve fibrinolytic enzyme production by *B. subtilis* WR350, a mutant derived from *B. subtilis* HQS-3^4^. First, culture medium containing inexpensive CSP was optimized in 250-mL shake flasks. Second, various OS conditions were evaluated for enhancing the production of fibrinolytic enzyme in a 100-L fermenter with the optimized medium. Finally, using the optimized and initial conditions, techno-economic assessments of the processes for fibrinolytic enzyme production were performed. The low-cost medium and suitable OS developed for fibrinolytic enzyme production by aerobic fermentation of *B. subtilis* WR350 may facilitate process optimization for the economical production of microbial fibrinolytic enzymes at an industrial scale.

## Materials and Methods

### Reagents

Bovine fibrinogen was purchased from Sigma (St. Louis, USA). Thrombin was provided by Hunan Yige Pharmaceutical Co., Ltd. (Xiangtan, China). Urokinase was provided by the National Institute for Food and Drug Control (Beijing, China). CSP was obtained from Shandong Kangyuan Bio-Tech Co., Ltd. (Yuncheng, China). Sucrose was purchased from Nanning Sugar Industry Co., Ltd. (Nanning, China). Cassava, corn, and wheat starches were purchased from local shops. Other reagents were of analytical grade and were commercially available.

### Bacterial strain

The *B. subtilis* strain WR350 used in this study was previously generated in our laboratory via UV mutagenesis of *B. subtilis* HQS-3^24^, which produced the same fibrinolytic enzyme as strain HQS-3^24^, and resulted in a significant improvement in fibrinolytic enzyme production. The previously described procedures for obtaining *B. subtilis* WR350 were as follows. A culture of *B. subtilis* HQS-3 was spread onto a preliminary screening agar plate containing 15 g/L casein and other essential nutrients. The plate was subsequently exposed to a germicidal lamp for 0–3 min, and the resulting mutant colonies were further screened on a Luria Bertani (LB) agar plate supplemented with 0.5 g/L fibrinogen and 0.5 U/mL thrombin. The stable mutant *B. subtilis* strain WR350 with the highest observed fibrinolytic activity was obtained and stored in equal volumes of LB broth and 50% (v/v) glycerol at −80 °C.

### Media and inoculum preparation

The media used in this work were prepared using the following methods. Seed medium was composed of 5 g glucose, 5 g yeast extract, 10 g tryptone, and 10 g NaCl per liter. The initial culture medium was composed of 20 g soy peptone, 50 g glucose, 2 g CaCl_2_, 3 g K_2_HPO_4_·3H_2_O, 1 g KH_2_PO_4_, and 3.5 g MgSO_4_·7H_2_O per liter. The carbon source control medium was composed of 20 g soy peptone, 2 g CaCl_2_, 3 g K_2_HPO_4_·3H_2_O, 1 g KH_2_PO_4_, and 3.5 g MgSO_4_·7H_2_O per liter. The pH values of all media were adjusted to 7.0 with 6 M HCl or 5 M NaOH. All media were sterilized in an autoclave (HVA-110, Japan) at 0.255 M Pa (approximately 115 °C) for 30 min.

Three hundred microliters of a glycerol stock of strain WR350 was inoculated into a 250-mL flask containing 50 mL of seed medium. The flask was incubated at 37 °C for 16 h with shaking at 160 rpm to prepare the inoculum. For all fermentation experiments, the inoculum size was maintained at 3% (v/v), and strain WR350 was incubated at 32 °C for 72 h.

### Medium optimization in shake flask

The one-variable-at-a-time approach was used to optimize the various nutrient parameters required for fibrinolytic enzyme production. Different carbon sources (20 g/L of glucose, sucrose, soluble starch, cassava starch, corn starch, or corn starch hydrolysate) were added to the carbon source control medium and evaluated for their ability to promote enzyme production, respectively. For each carbon source, the medium was supplemented with 20 g/L of various nitrogen sources (beef extract, yeast extract, peptone, urea, soybean meal (SBM), or CSP) for fermentation. With respect to the effects of inorganic salts on enzyme production, the carbon and nitrogen source were supplemented with 2 g/L of different inorganic salts (CaCl_2_, MgSO_4_·7H_2_O, MnSO_4_, CuCl_2_, ZnSO_4_, CrCl_2_, or KCl) for evaluation.

### Orthogonal experiment for medium optimization

The L_9_ (3^4^) orthogonal design, an orthogonal array of four factors with three levels, was used to further optimize the medium components. The assigned factors and levels were shown in Table 1, and experiments were performed in triplicate. Table 2 presented the experimental combinations and the resulting fibrinolytic enzyme production for each run. All experiments were performed in 250-mL flasks by varying the medium composition and culture conditions according to the orthogonal design (Table 2). The adopted variables (i.e., sucrose, CSP, and MgSO_4_·7H_2_O) were investigated for their effects on the fibrinolytic enzyme yield. Column A of the L_9_ (3^4^) orthogonal layout was left empty to calculate experimental error and evaluate the reliability of the experiment. The results were analyzed using SPSS version 20 for Windows (SPSS Inc., Chicago, IL, USA).

**Table 1:** Factors and levels of the L_9_ (3^4^) orthogonal experiment.

Parameters	Levels		
	1	2	3
A (Blank)	/	/	/
B (Sucrose)	25	35	45
C (CSP)	10	20	30
D (MgSO_4_·7H_2_O)	2	3	4

**Table 2:** Design and results of the L9 (3^4^) orthogonal experiment.

Factors	A	B	C	D	Fibrinolytic activity
	Blank	Sucrose	CSP	MgSO_4_·7H_2_O	U/mL
1	1	1	1	1	3454
2	1	2	2	2	4858
3	1	3	3	3	3820
4	2	1	2	3	4033
5	2	2	3	1	4756
6	2	3	1	2	2511
7	3	1	3	2	4052
8	3	2	1	3	2802
9	3	3	2	1	4395
K_1_	/	3846	2922	4202	
K_2_	/	4139	4429	3807	
K_3_	/	3576	4209	3552	
R	/	562	1506	649	

K_1_, K_2_, and K_3_ were the average fibrinolytic activity values corresponding to levels 1, 2, and 3 for each factor, respectively.

R = K_max_ − K_min_.

### Scale-up experiment

Batch fermentation was performed at 32 °C in a 100-L pilot fermenter (JINJIE Machinery, Henan, China) containing 50-L of the optimized medium using different agitation speeds (180, 200 and 220 rpm) and aeration rates (0.8, 1.0 and 1.2 vvm). Inoculum size was maintained at 3% (v/v), and all experiments lasted for 72 h. Dissolved oxygen (DO) level and pH were not controlled during fermentation, as the 100-L fermenter was not designed for DO measurements. The cell cultures were periodically harvested to evaluate fibrinolytic activity, biomass, pH, and the glucose and sucrose contents.

### Analytical methods

#### Determination of glucose and sucrose content

The glucose and sucrose contents were determined by high-performance liquid chromatography (HPLC) (LC-20 AB, Shimadzu, Japan) equipped with a Kromasil NH_2_ column (5 μm, 4.6 mm × 250 mm) and a refractive index detector. The mobile phase was composed of acetonitrile and water (v/v, 75:25) with a flow rate of 1 mL/min. The column temperature was maintained at 40 °C. The sample was adequately diluted with water, with 5 µL of each sample analyzed.

#### Fibrinolytic activity assay

Fibrinolytic enzyme activity was determined with the method described by Astrup and Mullertz^25^ using plasminogen-rich fibrin plates. Urokinase, which catalyzed the generation of plasmin from plasminogen, was used as a control. The dimension of the clear zone on each fibrin plate was measured, and fibrinolytic activity was calculated as per the standard curve generated using the control, where urokinase concentration (20–120 U/mL) was plotted against the transparent zone area.

#### Biomass determination

A convenient and most well-established method used to determine the growth state of a bacterial cell culture is to determine the optical density spectrophotometrically^26^. Thus, the growth of *B. subtilis* WR350 was determined by measuring the optical density at 600 nm (OD_600_).

#### Data analysis

All of the experiments were performed in triplicate, and the data presented were the means ± SD. The data were analyzed using SPSS version 20 for Windows (SPSS Inc., Chicago, IL, USA).

## Results and Discussion

### Optimization of significant nutritional factors affecting fibrinolytic activity

#### Optimization of carbon sources and concentrations

**Figure 1:**
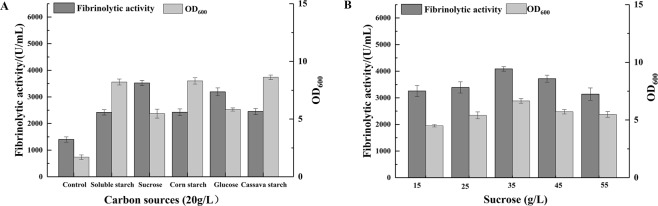
Effects of carbon sources (**A**) and different concentrations of sucrose (**B**) on fibrinolytic activity and biomass. The control medium contained 20 g/L soy peptone, 2 g/L CaCl_2_, 3 g/L K_2_HPO_4_·3H_2_O, 1 g/L KH_2_PO_4_, and 3.5 g/L MgSO_4_·7H_2_O (pH 7.0).

The carbon source is the most important raw material for microbial fermentation, providing the basic material for energy production and microbial components required for microbial fermentation^16^. The choice of carbon source has an important influence on the production of microbial enzymes. As shown in Fig. 1A, *B. subtilis* WR350 produced a small amount of fibrinolytic enzyme (1400 U/mL) from the carbon source control medium, whereas supplementation of this medium with the tested carbon sources (20 g/L) considerably improved fibrinolytic activity.

When sucrose was used as the carbon source, the highest fibrinolytic enzyme activity was observed (3523 U/mL), which was significantly higher than that obtained using cassava, corn, and soluble starches. The soluble starch resulted in the lowest fibrinolytic activity (2418 U/mL) (p < 0.01) (Fig. 1A). Compared to sucrose, the tested starches yielded higher biomasses and lower fibrinolytic activity. It was suggested that the tested starches were favored for cell growth of strain WR350 rather than fibrinolytic enzyme production. The tested sucrose and glucose not only maintained normal cell growth but also boosted enzyme production. Guangxi is the primary sugarcane- and sugar-producing area in China and produces approximately 60% of China’s sugarcane and sugar^27^. Our laboratory is located in Guangxi, China. The utilization of sucrose derived from local sugarcane with a low price for the cost-effective production of fibrinolytic enzyme, can stimulate local sales of sugar and promote economic development. Thus, sucrose was selected as the best carbon source. Similar results were obtained in a study by Vijayaraghavan *et al*.^28^, where sucrose was identified as the optimal carbon source for fibrinolytic enzyme production by *Bacillus* sp. IND12.

The effect of the sucrose concentration on fibrinolytic activity and biomass accumulation were investigated in 250-mL flasks, results were shown in Fig. 1B. It was clear that enzyme production was promoted by different sucrose concentrations, with the maximum activity (4086 U/mL) observed at a concentration of 35 g/L. Any further increase or decrease in sucrose concentration resulted in a decrease of fibrinolytic enzyme production. The results showed that high concentration of sucrose (>35 g/L) exerted a reduced amount of fibrinolytic enzyme production by *B. subtilis* WR350, which was probably due to substrate inhibition. Because of the positive effect on fibrinolytic enzyme production, 35 g/L of sucrose was included in the control medium for subsequent screening of key nitrogen sources.

#### Optimization of nitrogen sources and concentrations

**Figure 2:**
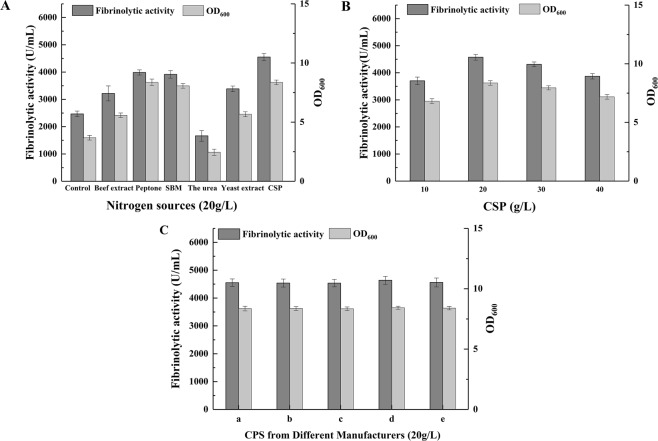
Effects of sources (**A**) and concentrations of nitrogen (**B**) and different types of CSP (**C**) on fibrinolytic activity and biomass. (**A**) The control medium contained 35 g/L sucrose, 2 g/L CaCl_2_, 3 g/L K_2_HPO_4_·3H_2_O, 1 g/L KH_2_PO_4_, and 3.5 g/L MgSO_4_·7H_2_O (pH 7.0). (**C**) Five types of CSP (represented by a–e) were obtained from Shandong Kangyuan Bio-Tech Co., Ltd. (Yuncheng, China), Shandong Dakai Bio-Tech Co., Ltd. (Jinan, China), Shandong Xinzhuoyuan Chemical Co., Ltd. (Jinan, China), Shanghai Yuantai Bio-Tech Co., Ltd. (Shanghai, China), and Zouping Juci Corn Biotechnology Bio-Tech Co., Ltd. (Binzhou, China), respectively.

The major nitrogen sources are important for microbial fermentation, as they are used to synthesize proteins, nucleic acids and other substances required for microbial growth. Thus, nitrogen sources are one of the most important raw materials for microbial fermentation^29^. As shown in Fig. 2A,B. subtilis WR350 produced a small amount of fibrinolytic enzyme (2420 U/mL) with a low biomass concentration in the nitrogen source control medium (35 g/L sucrose, 2 g/L CaCl_2_, 3 g/L K_2_HPO_4_·3H_2_O, 1 g/L KH_2_PO_4_, and 3.5 g/L MgSO_4_·7H_2_O). Interestingly, strain WR350 was able to produce fibrinolytic enzyme using the nitrogen-free control medium, which suggested that B. subtilis WR350 had the potential ability of nitrogen fixation. Actually, previously article had reported the ability of nitrogen fixation in B. subtilis^30^. But the detailed evidence about nitrogen fixation ability of B. subtilis WR350 should be further investigated in identification nitrogen fixation gene and ability of nitrogenase. Beef extract, yeast extract, peptone, urea, SBM, and CSP were individually added as nitrogen sources to the control medium for fermentation.

Figure 2A showed that the fibrinolytic activity of the control (2010 U/mL) was significantly lower than that of the experimental groups, with the exception of urea (1663 U/mL). Fibrinolytic enzyme production and cell growth were inhibited by urea, which may have been due to a lack of essential amino acids, lipids, or vitamins in the medium. When CSP was used as nitrogen source, the highest fibrinolytic enzyme activity (4552 U/mL) and the biomass concentration were observed. The fibrinolytic enzyme activity observed similarly when peptone (3987 U/mL) and SBM (3916 U/mL) were used as nitrogen sources, both of which were significantly higher than that observed using beef extract (3219 U/mL) and yeast extract (3387 U/mL) (*p* < 0.01) (Fig. 2A). Compared with the low-cost CSP and SBM, beef extract, yeast extract and peptone were excluded from the optimized medium due to their relatively higher price and lower fibrinolytic enzyme yields. Since SBM yielded lower fibrinolytic activity and was less commercially available than CSP, CSP was chosen as the optimal nitrogen source. As a byproduct of the corn wet-milling industry, CSP was an excellent source of nitrogen for most microorganisms because it was abundant in amino acids and polypeptides, and had considerable amounts of B-group vitamins^29,31^. These results showed the potential of CSP as a cheap nitrogen source to replace the costly soy peptone for fibrinolytic enzyme production. The stimulatory effects of CSP on enzyme production by *B. subtilis* WR350 were also observed in *Streptococci*^32^ and *Pleurotus eryngii*^33^.

The effects of the CSP concentration on fibrinolytic enzyme production and biomass accumulation were studied in shake flasks, results were shown in Fig. 2B. The maximum yields of fibrinolytic enzyme (4552 U/mL) and biomass were obtained using 20 g/L CSP. Because any further increase or decrease in CSP concentration resulted in a decrease in fibrinolytic enzyme production, 20 g/L of CSP was included in the control medium for subsequent screening of key inorganic salts.

To determine whether the cost-effective production of fibrinolytic enzyme depends on the use of a specific CSP, we investigated the effects of different types of CSP (purchased from different manufacturers in China) on fibrinolytic enzyme production (Fig. 2C). The results showed that the use of CSP from different origins resulted in similar fibrinolytic activity and biomass (*p* < 0.05), which could be due to similarities in the types and concentrations of essential nutrients present in these substrates. Therefore, the initially assayed CSP (Shandong Kangyuan Bio-Tech Co., Ltd., Yuncheng, China) was used in subsequent experiments.

#### Optimization of inorganic salts and concentrations

Inorganic salts are used to generate enzymes and also function as cofactors and regulators of enzyme activity centers^34^. In this experiment, the fermentation medium without any inorganic salt was used as a blank control (35 g/L sucrose and 20 g/L CSP, pH 7.0).

**Figure 3:**
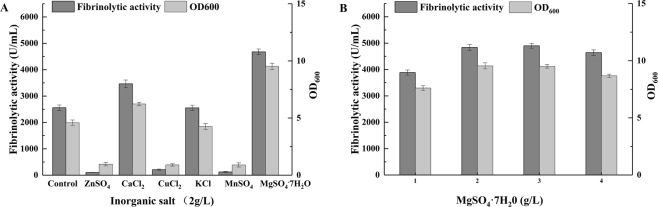
Effects of inorganic salts (**A**) and different concentrations of MgSO_4_·7H_2_O (**B**) on fibrinolytic activity and biomass. The control medium contained 35 g/L sucrose and 20 g/L CSP (pH 7.0).

As shown in Fig. 3A, compared with the control group, the addition of Mn^2+^ (124.33 U/mL), Cu^2+^ (209.88 U/mL), and Zn^2+^ (105 U/mL) decreased fibrinolytic activity and biomass. Additionally, the addition of Ca^2+^ (3830 U/mL), Mg^2+^ (4576 U/mL), and K^+^ (3652 U/mL) had a significant positive effect on fibrinolytic enzyme production (*p* < 0.01) (Fig. 3A). Pillai *et al*.^35^ reported that Mg^2+^ increased enzyme production of *B. subtilis* P13. Since the positive effect of Mg^2+^ on fibrinolytic activity was much higher than of the other tested inorganic salts, the addition of inorganic salts other than MgSO_4_·7H_2_O is not economical. Thus, MgSO_4_·7H_2_O was selected for further optimization.

As shown in Fig. 3B, the fibrinolytic enzyme yield was enhanced with the increasing concentration of MgSO_4_·7H_2_O. The highest yields of fibrinolytic enzyme (4959 U/mL) and biomass were obtained at a MgSO_4_·7H_2_O concentration of 2 g/L, which was the same as that obtained at a concentration of 3 g/L. Thus, 2 g/L of MgSO_4_·7H_2_O was added to the medium as the optimum inorganic salt.

### Orthogonal experiment

The experimental conditions for each run were listed in Table 2, with the enzyme yields shown in the last column. According to the R values (Table 2), the order of factors with respect to their contribution to fibrinolytic enzyme production was observed to be CSP > MgSO_4_·7H_2_O > sucrose. Furthermore, through analysis of variance (Table 3), the most important factor contributing to the product yield was observed to be factor B (CSP) (*p* < 0.05), followed by factor C (MgSO_4_·7H_2_O) and factor D (sucrose). To confirm the optimum combination (B_2_C_2_D_1_), three parallel experiments were performed using optimal concentrations of nutrients (35 g/L sucrose, 20 g/L CSP, and 2 g/L MgSO_4_·7H_2_O) according to range analysis presented in Table 3. The results of the orthogonal experiment were consistent with those of the single factor experiments, with an observed fibrinolytic enzyme activity in the optimized medium of 4961 U/mL.

**Table 3:** Analysis of variance.

Factors	SS	DOF	MS	*F* ratio	*P*
B (Sucrose)	475489.534	2	237744.049	2.906	0.256
C (CSP)	3973636.662	2	1986725.219	24.284	0.040*
D (MgSO_4_·7H_2_O)	643049.007	2	321559.323	3.930	0.203
Error	163633.56	2	81835.822		

SS: the sum of square deviation; DOF: the degree of freedom; MS: mean square.

*Significant at *P* < 0.05.

### 100-L fermenter scale-up experiment

**Figure 4:**
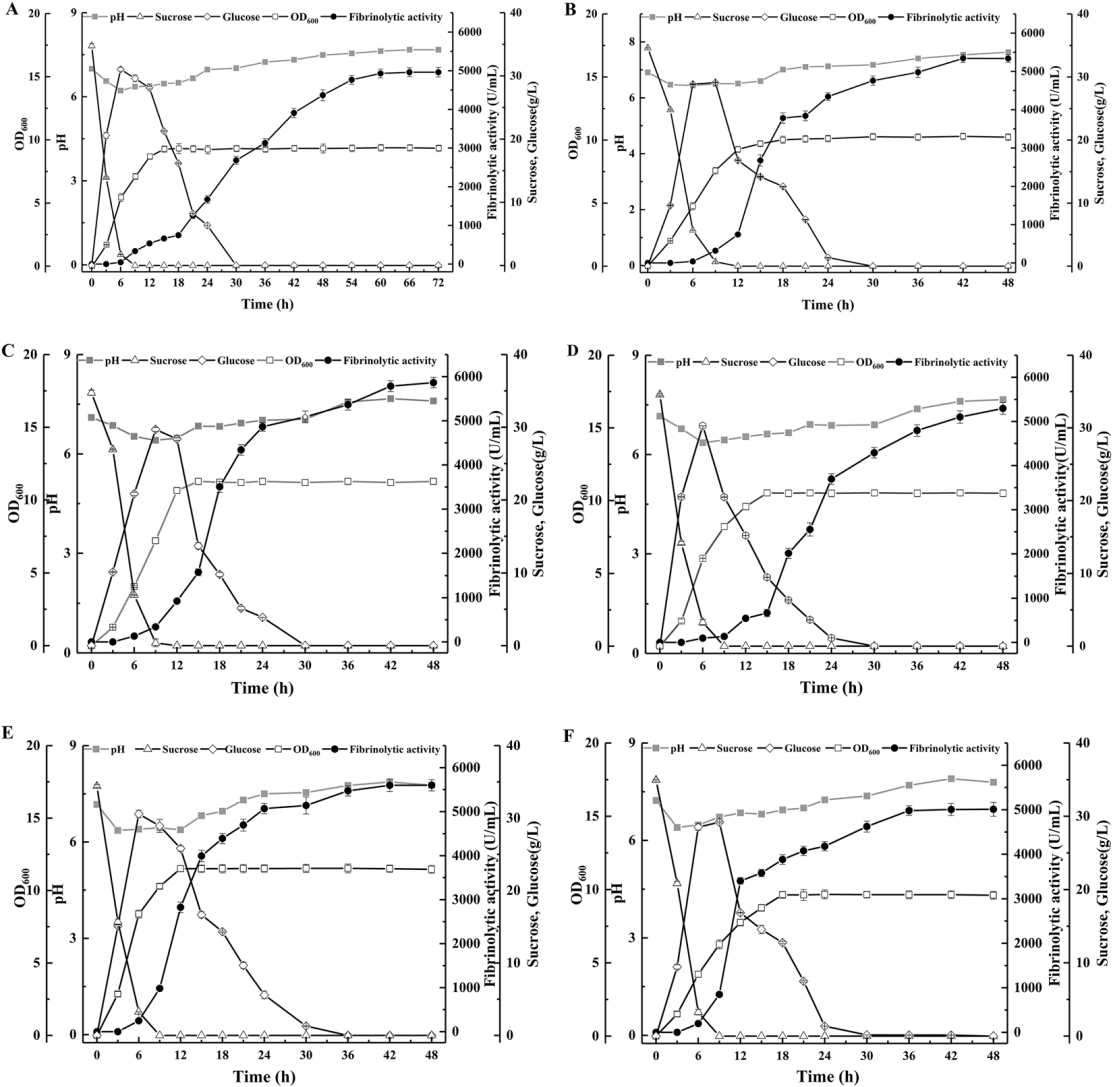
Time courses of batch fermentation and fibrinolytic enzyme activity obtained with the optimized medium by *B. subtilis* WR350 in a 250-mL flask (**A**) and a 100-L fermenter (**B–F**). (**B–D**) A constant aeration rate of 1.0 vvm was used with different agitation speeds of 180, 200, and 220 rpm, respectively. (**E,F**) A constant agitation speed of 200 rpm was used with different aeration rates of 1.2 and 0.8 vvm, respectively. The 100-L fermenter was not designed for on-line monitoring of DO.

A sufficient supply of oxygen is important for the growth and metabolism of *B. subtilis*^36^. If the oxygen demand of the cells is not satisfied, cell growth and aerobic synthesis of fibrinolytic enzyme will be inhibited^21^. To evaluate the effects of OS on enzyme production at the fermenter scale, batch fermentation with the optimized medium was conducted at different agitation speeds and aeration rates. Time course batch fermentation assays in 250-mL flasks was performed by incubating strain WR350 at 32 °C and 180 rpm (Fig. 4A), with subsequent scale-up experiments using various OS values sequentially performed in a 100-L fermenter at 32 °C (Fig. 4B–F).

Agitation helps to mix oxygen, heat, and nutrients in a fermenter efficiently, and the dispersal of air into small bubbles prevents bacteria from clustering and increases the gas-liquid contact area and dissolved oxygen level^37^. However, excessive agitation increases the power consumption and creates heterogeneous mixing and shear forces that can damage fragile microorganisms and affect product formation^20^. In contrast, when the agitation speed is too low, the viscosity of the fermentation medium will increase, leading to a reduction in mass transfer efficiency^38^. By maintaining the aeration rate constant at 1.0 vvm, the effects of agitation speed on fibrinolytic enzyme production were studied at 180, 200, and 220 rpm. As shown in Fig. 4B–D, the fibrinolytic enzyme activity increased in a similar manner at different agitation speeds before 9 h, after which the pattern of fibrinolytic enzyme production at the three agitation speeds differed. It was evident that at the same agitation speed of 180 rpm, faster enzyme production was obtained in the 100-L fermenter (Fig. 4B) compared to that observed in the 250-mL flask (Fig. 4A). This phenomenon may be associated with differences in OS^39^. In the 100-L fermenter, the maximum fibrinolytic enzyme activity (5865 U/mL) was observed at 200 rpm, with lower fibrinolytic enzyme yields observed at 180 rpm (5337 U/mL) and 220 rpm (5293 U/mL). Thus, the optimum agitation speed was determined to be 200 rpm, which was used for the further optimization of aeration rate.

Aeration provides oxygen for aerobic fermentation and facilitates the elimination of exhaust gas generated during the fermentation process^40^. An adequate OS is necessary for microbial growth during aerobic fermentation, but some microorganisms may be affected by oxygen toxicity at an excessive oxygen concentration^37^. Based on the agitation speed optimization results, the effects of aeration rate on fibrinolytic enzyme production were further evaluated by maintaining an agitation speed at 200 rpm. As shown in Fig. 4(C,E,F), the maximum fibrinolytic enzyme activity (5865 U/mL) was obtained at 1.0 vvm, with lower fibrinolytic enzyme activities observed at 0.8 vvm (5601 U/mL) and 1.2 vvm (5012 U/mL).

*B. subtilis* WR350 is a strictly aerobic microorganism. Thus, moderate aeration rates and agitation speeds can improve the growth of this microorganism. The effects of aeration rate and agitation speed on biomass were shown in Fig. 4. As shown in Fig. 4B–F, strain WR350 entered stationary phase at 15 h when batch fermentation was conducted at a constant aeration rate of 1.0 vvm with different agitation speeds. The highest biomass was obtained at agitation speed of 200 rpm, but the biomass was reduced at an agitation speed of 220 rpm, probably owing to shear force and mixing effects. A high agitation speed may cause high shear stress, which negatively influences cell activities in the fermentation system. Aeration rate is an important factor for the growth of aerobic microorganisms and can play an important role in scale-up of aerobic biosynthesis systems. At a constant agitation speed of 200 rpm, the biomass concentration increased as the aeration rate increased from 0.8 to 1.2 vvm (Fig. 4(C,E,F)). The highest biomass was obtained at the maximum aeration rate of 1.2 vvm. However, the highest fibrinolytic activity was observed at 1.0 vvm, which corresponded with a lower biomass. Thus, cell growth and product formation of the aerobic strain WR350 appear to be associated with both aeration rate and agitation speed. In addition, a further increase in the aeration rate to 1.2 vvm in the 100-L fermenter (Fig. 4E) yielded the highest biomass but relatively less fibrinolytic enzyme, which may be associated with an excessive OS that favors the depletion of nutrients for biomass accumulation. An excess of oxygen during fermentation may also result in the direct oxidation and loss of substrate, which decreased productivity. Given the above results, an agitation speed of 200 rpm and an aeration rate of 1.0 vvm were determined to be ideal for batch fermentation in a 100-L fermenter, providing a suitable environment for the aerobic synthesis of fibrinolytic enzyme.

In all culture systems, the pH decreased slightly during the first 48 h of the fermentation from an initial value of 7.0 to 6.4 and then slowly increased to 7.7 at the end of the fermentation (Fig. 4). Organic acids produced by the metabolism of sucrose during the exponential phase of strain WR350 may be the reason for the decrease in pH. The pH increased when sucrose was completely consumed during the stationary phase probably due to the production of ammonia from CSP in the fermentation medium.

The HPLC results showed that the initial sucrose content of the fermentation medium was 35 g/L, and the sucrose was completely hydrolyzed at 9 h. These results suggested that *B. subtilis* WR350 first converted sucrose into glucose and fructose, after which it used the hexoses for cell growth and fibrinolytic enzyme production. The hexose content in the 250-mL flask and 100-L fermenter assays reached the maximum at 6 and 9 h respectively, with the hexoses exhausted by 30 h in both systems (Fig. 4).

At both the flask and fermenter scale (Fig. 4), most of the fibrinolytic enzyme was synthesized during stationary phase. Extracellular protease production is considered to be a manifestation of nutrient limitation at the onset of stationary phase^41^. In the present study, when cells entered stationary phase, sucrose was exhaustively consumed. Thus, nutrient deficiency and sufficient OS may have induced the aerobic synthesis of fibrinolytic enzyme by *B. subtilis* WR350, which could efficiently hydrolyze the complex carbon-nitrogen sources present in CSP into easy-use carbon sources and other essential nutrients for cell survival. Compared to batch fermentation in flasks, better OS and mixing in the fermenter may have been responsible for the higher productivity observed during stationary phase.

### Fermentative benefits evaluation

The fermentative production of fibrinolytic enzyme by strain WR350 was successfully scaled up to a 100-L fermenter, which resulted in an enhanced fibrinolytic activity (5865 U/mL at 42 h) (Fig. 4C) compared to that observed in the 250-mL flask (4930 U/mL at 60 h) (Fig. 4A). In addition, under the same environmental conditions, batch fermentation using the initial medium in the 100-L fermenter resulted in a lower fibrinolytic activity (4500 U/mL) than that obtained using the optimized medium. Considering the microbial production of fibrinolytic enzyme under submerged fermentation^37,42–44^, the optimum yield obtained in the present study is among the highest described in the literature to date (Table 4).

**Table 4:** Comparison of fibrinolytic enzyme production under submerged fermentation.

Strains	Medium components	Fermentation systems	Yield (U/mL)^a^
*B. cereus* SRM-001^43^	Corn flour, soybean powder, MnSO_4_	500-mL flask	1450
*B. subtilis* DC-2^42^	Soy peptone, maltose, yeast extract, K_2_HPO_4_, NaH_2_PO_4_, CaCO_3_	500-mL flask	1223.61
*B. subtilis* D21-8^37^	Cassava starch, SBM, CaCl_2_	100-L fermenter	3787
*B. subtilis* LD-8547^44^	Rice power, soybean power, NH_4_NO_3_, CaC1_2_, MgSO_4_, K_2_HPO_4_, KH_2_PO_4_	15-L fermenter	4220
*B. subtilis* WR350 (this study)	Initial medium	100-L fermenter	4500
	Optimized medium	100-L fermenter	5865

^a^Determined by the fibrin plate method.

As shown in Table 5, fermentation costs were compared between the initial and optimized medium in the 100-L fermenter. All of the feedstock and energy prices were provided by local suppliers. In the present study, the total fermentation costs of production of fibrinolytic activity per 10^9^ U using the initial and optimized medium were $59.04 and $13.78, respectively. As the optimized medium was composed of inexpensive sucrose and CSP, the medium cost was reduced by 91.5%. In addition, the reduction in steam used for sterilization was 52 kg per 10^9^ U fibrinolytic activity when using the optimized medium compared to the initial medium. In addition, replacing expensive soy peptone and glucose with cheap CSP and sucrose resulted in a shorter fermentation period and lower energy consumption. Thus, the results of this study provided an efficient and simple strategy to produce fibrinolytic enzyme in a 100-L pilot fermenter using sucrose and CSP as low-cost substrates, which reduced fermentation costs by 76.66% compared to that using the initial medium.

**Table 5:** Comparison of the cost between the optimized and initial medium to produce 10^9^ U of fibrinolytic activity in a 100-L fermenter.

Parameter	Medium		Unit price ($)^a^
	Initial	Optimized	
Glucose	11.15 kg	N	0.29 kg^−1^
Sucrose	N	5.985 kg	0.26 kg^−1^
Soy peptone	4.46 kg	N	5.91 kg^−1^
CSP	N	3.42 kg	0.35 kg^−1^
MgSO_4_·7H_2_O	0.781 kg	0.342 kg	0.12 kg^−1^
CaCl_2_	0.446 kg	N	0.12 kg^−1^
KH_2_PO_4_	0.223 kg	N	3.45 kg^−1^
K_2_HPO_4_·3H_2_O	0.669 kg	N	3.45 kg^−1^
Medium cost	$ 32.82	$ 2.79	
Steam	223 kg	171 kg	0.023 kg^−1^
Energy for fermentation	2.4 kW·h	2.4 kW·h	0.07 kW^−1^·h^−1^
Fermentation period	72 h	42 h	
Fibrinolytic activity	4500 U/mL	5865 U/mL	
Total fermentation cost	$59.04	$13.78	

N: No addition.

^a^Available in November 2018.

## Conclusions

The results of this study showed that CSP and sucrose may serve as low-cost substrates for the economical production of fibrinolytic enzyme in a 100-L fermenter by *B. subtilis* WR350. Strain WR350, which exhibited high fibrinolytic activity, showing great potential in industrial applications. Agitation speed and aeration rate significantly affected the synthesis of fibrinolytic enzyme by *B. subtilis* WR350. The efficient control of agitation speed and aeration rate in a well-designed fermenter was recommended for improving the productivity of fibrinolytic enzyme in the future. Furthermore, the techno-economic evaluation confirmed the effectiveness of using sucrose and CSP instead of glucose and soy peptone for fibrinolytic enzyme production.
